# Combining two Meishan F2 crosses improves the detection of QTL on pig chromosomes 2, 4 and 6

**DOI:** 10.1186/1297-9686-42-42

**Published:** 2010-11-25

**Authors:** Flavie Tortereau, Hélène Gilbert, Henri CM Heuven, Jean-Pierre Bidanel, Martien AM Groenen, Juliette Riquet

**Affiliations:** 1 INRA, UMR 0444 Génétique Cellulaire, F-31326 Castanet-Tolosan, France; 2 INRA, UMR 1313 Génétique Animale et Biologie Intégrative, F-78352 Jouy-en-Josas, France; 3 Wageningen University, Animal Breeding and Genetics Group, 6700 AH Wageningen, The Netherlands

## Abstract

**Background:**

In pig, a number of experiments have been set up to identify QTL and a multitude of chromosomal regions harbouring genes influencing traits of interest have been identified. However, the mapping resolution remains limited in most cases and the detected QTL are rather inaccurately located. Mapping accuracy can be improved by increasing the number of phenotyped and genotyped individuals and/or the number of informative markers. An alternative approach to overcome the limited power of individual studies is to combine data from two or more independent designs.

**Methods:**

In the present study we report a combined analysis of two independent design (a French and a Dutch F2 experimental designs), with 2000 F2 individuals. The purpose was to further map QTL for growth and fatness on pig chromosomes 2, 4 and 6. Using QTL-map software, uni- and multiple-QTL detection analyses were applied separately on the two pedigrees and then on the combination of the two pedigrees.

**Results:**

Joint analyses of the combined pedigree provided (1) greater significance of shared QTL, (2) exclusion of false suggestive QTL and (3) greater mapping precision for shared QTL.

**Conclusions:**

Combining two Meishan x European breeds F2 pedigrees improved the mapping of QTL compared to analysing pedigrees separately. Our work was facilitated by the access to raw phenotypic data and DNA of animals from both pedigrees and the combination of the two designs with the addition of new markers allowed us to fine map QTL without phenotyping additional animals.

## Background

Over the past fifteen years, the construction of genetic maps in livestock species has enhanced efforts to dissect the molecular basis of the genetic variation of agriculturally important traits. In pig, a number of experiments have been set up to identify QTL and many chromosomal regions harbouring genes influencing traits of interest have been identified [1] and reported in QTLdb http://www.genome.iastate.edu/cgi-bin/QTLdb/index[2]. However, in most cases mapping resolution remains limited and the QTL detected are rather inaccurately located. Mapping accuracy can be improved by increasing the number of phenotyped and genotyped individuals and/or the number of informative markers. However, collecting this additional information is often time-consuming and/or expensive. An alternative approach to overcome the limited power of individual studies is to combine data from two or more independent designs. Combining several pedigrees together increases the number of animals without additional phenotyping or genotyping costs. Without access to raw data, meta-analysis of published results can be an informative approach to increase precision. Allison and Heo [3] have proposed meta-analytical techniques that can be used under difficult conditions. However, these analyses are complicated by the differences among testing methods and experimental designs and finally, the gain in accuracy of QTL mapping is limited. Availability of the raw data to analyse jointly independent data sets is probably a better way to combine different QTL mapping designs. In pig, some studies aiming at combining pedigrees in order to increase the power of QTL detection have already been carried out. Walling et al. [4] have combined French, British, Dutch, American, Swedish and German studies to detect QTL on pig chromosome 4 or SSC4 (for *Sus scrofa *chromosome 4) and Perez-Enciso et al. [5] have combined pedigrees from Spanish, French and German designs to refine the location of a QTL for growth and fatness traits on SSCX. However, these analyses are complicated by the lack of common markers and often by slight differences in trait definitions and measurements. In addition, parental populations are usually different, and the QTL segregating in the various designs are not necessarily the same. Under these conditions, combining data sets from different origins may not be optimal to improve estimations of QTL localisations and effects.

Here we report an analysis of QTL located on SSC2, SSC4 and SSC6, that combines French and Dutch F2 pedigrees involving Meishan (MS), Large White (LW) and Landrace (LR) breeds. The analysis was focused on these three pig chromosomes because previous detection analyses [6-12] have shown that the QTL identified on these chromosomes contribute less to the global variance of the traits than QTL detected for example on SSC7 or SSCX. To optimize this joint analysis and re-construct a unique genetic map from the 2000 F2 offspring of this combined design, additional microsatellite markers were included in either one or both pedigrees. Using single- and multiple-QTL mapping analyses on each pedigree and on the combined pedigree, we investigated the benefits of combining pedigrees (i.e. doubling the pedigree size) to refine the location of QTL for growth and fatness on SSC2, 4 and 6.

## Methods

### Pedigrees and phenotypic data

QTL mapping data from two experimental F2 crosses between European pig breeds x Meishan were used: (1) the French PORQTL pedigree produced at INRA [6], by mating six Large White sires and six Meishan dams and then six F1 sires and 20 F1 dams to produce 1052 F2 animals; all pigs were born and raised at the INRA GEPA experimental unit (Poitou-Charentes) and (2) a Dutch pedigree, obtained at the University of Wageningen (WU) [13,14] by mating 19 Meishan sires and 126 Large White or Landrace dams and then 39 F1 sires and 265 F1 dams to produce 1212 F2 offspring; this Dutch design was conducted in five different breeding companies. Among the 39 Dutch half-sib families, we selected the 24 largest families, amounting finally to 1919 French and Dutch F2 animals.

Details on the phenotypic data have been reported respectively for the French pedigree in [6] for growth and fatness traits, [15] for teat number, [8] for carcass composition traits and [7] for IntraMuscular Fat (IMF) and for the Dutch pedigree in [10-12] for growth, fatness and meat quality traits [10-12] and in [9] for teat number. Nine traits related to growth, fatness and teat numbers (Table 1) were included in a joint analysis of the pedigrees. Seven of these nine traits i.e. birth weight, weaning weight, carcass weight, teat number, IMF, Back-Fat Thickness (BFT) between the 3rd and 4th ribs of a carcass at 6 cm from the spine and *Longissimus Dorsi *(LD) depth were chosen because they had been recorded in both designs with the same conditions. The two remaining common traits i.e. Life Weight Gain (LWG) and meat percentage were already available for the Dutch pedigree and had to be computed for the French pedigree. Meat percentage was computed in the Dutch pedigree with the Hennessy Grading Probe formula taking BFT and muscle depth into account. For the French pedigree, we applied a similar formula as that used in France at the time of the experiment [16] which is also based on back fat thickness and muscle depth (meat percentage = 55.698 - 0.710 × BFT + 0.198 × LD). LWG is an average daily gain estimated throughout the entire animals' life and is calculated as weight/age.

**Table 1:** Studied traits in the French and Dutch pedigrees

Pedigree	Trait	N	Mean	SD
**French**	**birth weight (kg)**	**1052**	**1.25**	**0.27**
**French**	**weaning weight (kg)**	**1052**	**5.46**	**1.13**
**French**	**teat number**	**1046**	**14.82**	**1.56**
**French**	**carcass weight (kg)**	**529**	**59.11**	**10.48**
**French**	**BFT (mm)**	**521**	**17.00**	**5.00**
**French**	**LD depth (mm)**	**521**	**35.00**	**9.00**
**French**	**meat percentage (%)**	**521**	**50.44**	**3.66**
**French**	**LWG (kg/day)**	**960**	**0.49**	**0.10**
**French**	**IMF (%)**	**248**	**1.69**	**0.54**
French	X2 (mm)	521	17.80	5.22
French	shoulder weight (kg)	489	4.71	0.84
French	midriff weight (kg)	489	1.16	0.33
French	ham weight (kg)	489	5.79	0.92
French	loin weight (kg)	489	8.07	1.46
French	leaf fat weight (kg)	489	0.42	0.23
French	foot weight (kg)	489	1.03	0.22
French	belly weight (kg)	489	2.96	0.65
French	kidney weight (kg)	487	0.11	0.02
French	head weight (kg)	481	4.81	0.82

**Dutch**	**birth weight (kg)**	**867**	**1.22**	**0.22**
**Dutch**	**weaning weight (kg)**	**864**	**8.23**	**2.00**
**Dutch**	**teat number**	**869**	**15.42**	**1.20**
**Dutch**	**carcass weight (kg)**	**548**	**70.24**	**9.59**
**Dutch**	**BFT (mm)**	**565**	**22.19**	**5.70**
**Dutch**	**LD depth (mm)**	**563**	**40.82**	**6.72**
**Dutch**	**meat percentage (%)**	**565**	**48.53**	**4.25**
**Dutch**	**LWG (kg/day)**	**551**	**0.53**	**0.08**
**Dutch**	**IMF (%)**	**557**	**1.87**	**0.88**
Dutch	pH_24 (LD)	565	5.67	0.27
Dutch	L*	563	53.85	4.73
Dutch	a*	565	17.13	1.83
Dutch	b*	565	9.52	1.91
Dutch	pH_24 (ham)	565	5.83	0.32
Dutch	driploss (%)	563	2.68	1.52
Dutch	cookloss (%)	564	26.35	3.46
Dutch	shear force (N)	564	39.20	10.75

Traits indicated in bold are common to the two independent pedigrees; N = number of analysed F2 for the corresponding trait; SD = Standard Deviation; BFT = Back-Fat Thickness (referred as X4 in [8]); LD = *Longissimus Dorsi*; LWG = Life Weight Gain; IMF = IntraMuscular Fat content; X2 is another measurement of back-fat thickness; pH_24 = pH measured 24 h after slaughter; a* = meat redness; b* = meat yellowness; L* = meat lightness

Some additional economically important traits that had been recorded only in one of the two pedigrees but for which a significant QTL had been previously detected, were analysed only in the corresponding pedigree. These traits were related to additional fatness (X2, measured between the 3rd and the 4th lumbar vertebrae at 8 cm from the spine), cut weights (shoulder, midriff, ham, loin, leaf fat, foot, belly, kidney and head) for the French pedigree and meat quality (pH in m. *Longissimus Dorsi *and in m. *Semimembranosus *taken 24 h after slaughter, L*, a* and b* colour values of m. *Longissimus Dorsi*, driploss, cookloss and shear force) for the Dutch pedigree (Table 1). These traits are not shared by both designs, but are economically important and thus were re-analysed in this study with additional microsatellite markers.

### Genotyping

**Figure 1:**
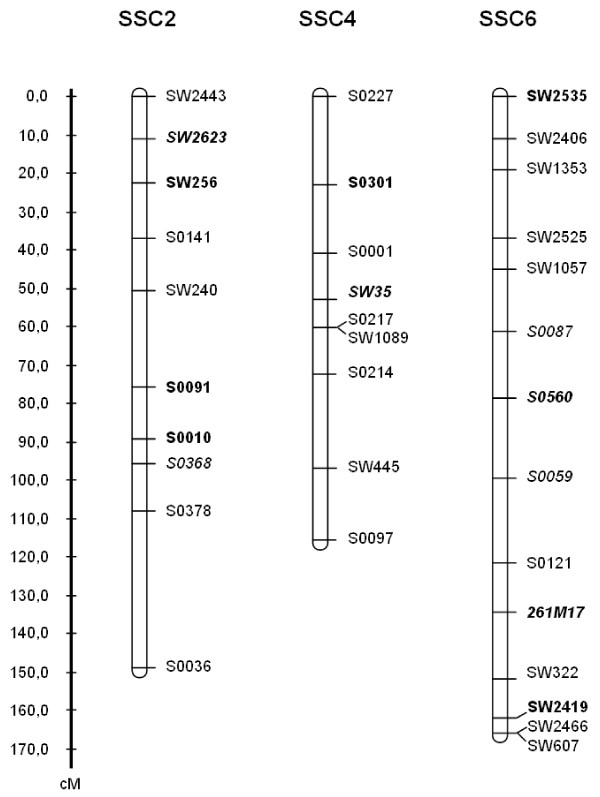
**Linkage maps of SSC2, SSC4 and SSC6 for the combined pedigree**. Microsatellite markers in bold were added for the analysis of the French animals and markers in italics for the Dutch animals; *: on SSC4, microsatellite S0217 is genotyped only on Dutch animals and SW1089 on French animals; on SSC6, SW2466 is genotyped only on Dutch animals and SW607 on French animals

In order to compare QTL detection results among the French, Dutch and combined pedigrees, a consensus linkage map based on genotyping data from the two Meishan x European breeds F2 populations was generated. The aim was to have a density of one marker every 10 cM within the previously described QTL regions and every 20 cM on the rest of the chromosomes. QTL regions extended from the telomere of the p arm to microsatellite SW240 on SSC2, between microsatellites S0301 and S0214 on SSC4 and along two regions on SSC6 (between SW2535 and SW1057 and between S0059 and SW607). Initially, French and Dutch pedigree were genotyped over these three chromosomes with 22 and 29 microsatellite markers respectively [6,11]. Five microsatellite markers on SSC2, five on SSC4 and six on SSC6 were common to both designs. Additional informative microsatellite markers were included for one or both pedigree(s), to obtain a unique set of common markers. Among the markers genotyped on both pedigrees on SSC6, microsatellite 261M17 was specifically designed from the BAC end-sequence bT261M17SP6 with primers 5'-CTCTTCCATTCCCTGATTGC-3' and 5'-CCCCTTCCTCACCTCTTTCT-3' to fill the gap between S0121 (122 cM) and SW322 (152 cM). On the common map, this new microsatellite is located 12.8 cM from S0121 and 17.4 cM from SW322. Finally, for SSC2 four additional microsatellites were analysed in the French pedigree and two in the Dutch population, for SSC4, two in the French pedigree and one in the Dutch population and for SSC6, four in the French pedigree and three in the Dutch population. New genotyping data were obtained at INRA as previously described [6]. All the genotypes were validated and stored in the Gemma database [17]. Only common markers were kept in the analysis, except for S0217 and SW2466 that were used only on the Dutch animals and SW1089 and SW607 only on the French animals. Microsatellites S0217 and SW1089 and microsatellites SW2466 and SW607 which mapped to the same position respectively on SSC4 and SSC6 were considered as unique markers in the combined analysis. Marker maps were ordered using CriMap package [18], considering all the F2 animals of the two designs. The sex-averaged maps are presented in Figure 1.

### Statistical analyses

Before QTL detection, phenotypes were corrected for the usual fixed effects using a linear model (GLM procedure, SAS^® ^9.1, SAS Institute, Inc.). For traits previously analysed, published fixed effects and covariates were used and for the other traits, fixed effects and covariates that were significant at the 5% level in the variance analysis were kept in the final model. Corrected data showed similar variances for the traits common in both designs but recorded independently so that no standardisation was applied.

QTL analysis using these corrected data was performed with the QTLMap software developed at INRA [19,20] based on interval mapping without any hypothesis on the number of QTL alleles present in the Meishan and European breeds.

The test statistic was computed as the ratio of likelihoods under the hypothesis of one (H1) vs. no (H0) QTL linked to the set of markers considered. Under hypothesis H1, a QTL effect for each sire and each dam (only dams with more than 10 offspring were taken into account) was fitted to the data. All sufficiently probable (above 0.10) dam phases were considered, so that the likelihood *Λ *could not be entirely linearised. For every cM along a linkage group, the likelihood *Λ *could be written as:

$$\Lambda^{x}=\prod_{i,j}\sum_{hd_{ij}}p(hd_{ij}|M_{i},\overset{\wedge }{h}s_{i})\prod_{ijk}f({\tilde{y}}_{ijk}|\overset{\wedge }{h}s_{i},hd_{ij},M_{i})$$

where: ∏_*i, j *_is a product over full-sib families of sire *i *and dam i*j*, $\sum_{hd_{ij}}$ is a summation over dam phases *hd*_*ij *_with a probability greater than 0.10, $\overset{\wedge }{h}s_{i}=\arg\max_{hs_{i}}p(hs_{i}|M_{i})$, *p*(*hs*_*i*_|*M*_*i*_) = linkage phase *hs*_*i *_probability for sire *i *given marker information *M*_*i*_, $p(hd_{ij}|M_{i},\overset{\wedge }{h}s_{i})$ = linkage phase for dam *ij *given marker information *M*_*i *_and sire linkage phase, $f({\tilde{y}}_{ijk}|\overset{\wedge }{h}s_{i},hd_{ij},M_{i})$ = density function of the adjusted phenotype ${\tilde{y}}_{ijk}$ of the offspring *ijk *of the *ij*th dam and the *i*th sire, conditional on the chromosome segments transmitted by the sire (*q*_*s*_) and the dam (*q*_*d*_). ${\tilde{y}}_{ijk}$ is supposed to be normally distributed with a mean $\sum_{qs=1}^{2}\sum_{qd=1}^{2}p(d_{ijk}^{x}=(q_{s},q_{d})|hs_{i},hd_{ij},M_{i})(\mu_{i}^{xqs}+\mu_{ij}^{xqd})$ and a variance *σ*^*2*^_*i *_estimated within each sire family, where $p(d_{ijk}^{x}=(q_{s},q_{d})|hs_{i},hd_{ij},M_{i})$ is the transmission probability from parents *i *and *ij *to offspring *ijk*, and $\mu_{i}^{xqs}$ and $\mu_{ij}^{xqd}$ can be parameterised as $\mu_{i(j)}^{x1}=\mu_{i(j)}+\alpha_{i(j)}^{x}/2$ and $\mu_{i(j)}^{x2}=\mu_{i(j)}-\alpha_{i(j)}^{x}/2,\alpha_{i}^{x}$ and $\alpha_{ij}^{x}$ being the within-half-sib and within-full-sib average QTL substitution effects and *μ*_*i(j) *_being the family mean for parent *i(j)*. Average substitution effects, which in the present case are equivalent to additive values (*a*), were hence estimated within each sire family as $\mu_{i}^{x1}-\mu_{i}^{x2}$ and within each dam family as $\mu_{ij}^{x1}-\mu_{ij}^{x2}$, and averaged over families to estimate the average QTL effect in the population.

To guarantee an accurate estimation of the sire QTL effects, only sire families with more than 30 progeny were retained in the analysis, thus 15 sire families were omitted from the Dutch pedigree. Due to number of progeny per dam, dam effects were estimated for all dams in the French pedigree, whereas none was estimated in the Dutch pedigree.

The maximum LRT along the linkage group indicated the most likely position for a QTL. Significance thresholds were empirically computed using 1000 simulations under the null hypothesis, assuming an infinitesimal polygenic model (i.e. the trait is controlled by an infinite number of additive loci, each with infinitesimal effect and is thus not influenced by a major QTL) and a normal distribution of performance traits [21]. In practice, for progeny p, simulated phenotypes y_p _were sampled as the sum of a polygenic part u_p _and an environmental part e_p _with normal distributions of mean = 0 and variances depending on the heritability of the trait, the total phenotypic variance being 1. The polygenic parts were sampled on the F1 sires and dams (u_s _and u_d_, respectively) and the transmission to the progeny was simulated as u_p _= 0.5 u_s _+ 0.5 u_d_, resulting in y_p _= 0.5 u_s _+ 0.5 u_d _+ e_p _for progeny p. Simulations were preferred to permutations because of the family structures [22]. With these structures, permutations have to be performed within full-sib families to respect data variability in the different families. In our case, data number within each family was not sufficient to achieve an extensive description of the distribution of the test statistic under the null hypothesis. QTL were considered significant if the chromosome-wide significance threshold exceeded 5% and suggestive if it exceeded 10%. Chromosome-wide significance thresholds were preferred to genome-wide significance thresholds since only three chromosomes were included in the analyses. Estimated significant thresholds (at the 5% chromosome-wide level) varied with traits and pedigree ranging between 45 and 50 for each independent pedigree and 80 and 85 for the combined pedigree. Confidence intervals around a QTL position were empirically determined by the "2-LOD drop-off" method [23].

For each chromosomal region, QTL detection analyses were applied separately on the French and Dutch pedigrees, and then on the combination of both pedigrees thereafter named "combined pedigree".

Additional analyses were carried out with QTLMap to test the segregation of two linked QTL in a linkage group [24] and revealed two situations: (1) when a significant QTL had been previously detected (H0 versus H1), the null hypothesis was "one QTL segregating at the maximum likelihood position estimated under H1" and (2) when no QTL had been previously detected (H0 versus H1), the null hypothesis was "no QTL". In both cases, the alternative hypothesis (H2) was "two linked QTL segregating on the linkage group". The LRT were computed following a grid-search strategy, using first 5 cM steps along the chromosome to identify significant regions in which then finer steps (1 cM) were applied. Significance thresholds were empirically estimated by a thousand simulations under the null hypothesis as described by Gilbert and Le Roy [25]. When H0 was "no QTL", thresholds were the same as those computed previously for single QTL tests. When H0 was "one QTL segregating at the maximum likelihood position x_max estimated under H1", simulations were done assuming that the trait was controlled by a QTL located at x_max and having the effect estimated for the maximum likelihood at position x_max in the single QTL analysis, all F1 being considered as heterozygous for the QTL. Estimated significant thresholds (at the 5% chromosome-wide level) varied with traits and pedigree, ranging between 85 and 90 for each independent pedigree and between 140 and 150 for the combined pedigree.

Finally, QTL detection was also carried out on the adjusted data using a Line-Cross model (LC) with the online version of QTLExpress [26]. In this report, the method is only briefly described since results are not shown in detail. The LC model assumed that Meishan and European breeds were fixed for alternate QTL alleles. With the LC model, the adjusted phenotypes were fitted to a linear model including additive and dominant components [27] and the chromosome-wide significance thresholds were determined by permutations of data as described by Churchill and Doerge [28].

## Results

### Linkage maps

For chromosomes SCC2, SSC4 and SSC6, the estimated marker orders of their linkage maps were consistent with those of the published USDA-MARC linkage maps http://www.marc.usda.gov/genome/swine/swine.html and their sex-averaged lengths were 149 cM, 116 cM and 166 cM, respectively (Figure 1).

### QTL detection

Table 2 shows the QTL detection results for each chromosome separately and for each independent pedigree and the combined pedigree. These results were obtained by using the half-full sib model with the QTLMap software. Additional analyses were done with the Line Cross model (which assumes that parental breeds are fixed for alternate QTL alleles) with the QTLExpress online Software [26]. The same QTL were detected with the HFS and LC models (data not shown), except for the QTL underlying birth weight that was only described with the HFS model in the combined pedigree. Moreover, it is worth noting that with LC model, most QTL were significant at a 1% chromosome-wide threshold whereas with HFS model significant QTL were detected at different thresholds (Table 2).

**Table 2:** QTL detected in the two independent and the combined pedigrees by the single QTL detection analysis with significance level <10%

Pedigree	Trait	SSC	Position (cM)	LRT	Threshold	Number of segregating sires	QTL effect	References
**French**	**BFT**	**2**	**0 [0-8]**	**47.2**	**+**	**2**	**+ 0.25**	[8]
French	loin	2	0 [0-3]	58.4	**	3	- 0.30	[8]
**French**	**IMF**	**4**	**0 [0-12]**	**37.0**	**+**	**3**	**- 0.009**	**/**
**French**	**BFT**	**4**	**61 [55-66]**	**45.5**	**+**	**4**	**+ 0.19**	[8]
**French**	**LWG**	**4**	**66 [56-71]**	**69.6**	**	**2**	**- 0.18**	[6]
French	x2	4	14 [6-23]	47.0	+	2	- 0.006	/
French	head weight	4	42 [17-49]	44.6	+	3	- 0.25	/
French	birth weight	4	53 [45-58]	61.3	**	4	- 0.07	[6]
French	belly weight	4	55 [48-61]	56.9	**	2	- 0.04	[8]
**French**	**teat number**	**6**	**110 [98-130]**	**46.0**	**+**	**3**	**+ 0.03**	[15]
French	loin weight	6	99 [92-108]	46.6	+	3	+ 0.22	/
French	midriff weight	6	144 [137-150]	59.1	**	4	- 0.15	/

**Dutch**	**weaning weight**	**2**	**21 [17-24]**	**46.2**	**+**	**5**	**+ 0.03**	**/**
**Dutch**	**BFT**	**2**	**28 [18-46]**	**60.4**	**	**9**	**+ 0.22**	[11]
**Dutch**	**meat**	**2**	**30 [19-47]**	**63.8**	**	**8**	**- 0.31**	**/**
Dutch	a*	2	26 [19-36]	51.2	*	10	+ 0.02	[10]
**Dutch**	**IMF**	**4**	**0 [0-6]**	**47.0**	**+**	**9**	**+ 0.14**	[11]
**Dutch**	**birth weight**	**6**	**136 [130-144]**	**47.4**	**+**	**8**	**- 0.13**	**/**
**Dutch**	**teat number**	**6**	**154 [146-161]**	**47.5**	**+**	**8**	**- 0.05**	**/**
Dutch	L*	6	103 [91-118]	61.3	**	7	+ 0.20	/

Combined	BFT	2	1 [0-5]	95.6	*	12	+ 0.34	
Combined	meat	2	32 [25-46]	97.8	**	10	- 0.32	
Combined	IMF	4	0 [0-5]	84.0	*	12	+ 0.11	
Combined	teat number	4	46 [37-51]	75.7	+	10	- 0.04	
Combined	birth weight	4	53 [49-57]	82.1	*	9	- 0.10	
Combined	LWG	4	83 [59-90]	98.6	**	11	- 0.30	
Combined	teat number	6	104 [94-113]	84.8	*	11	- 0.01	

Traits indicated in bold are common to the two independent pedigrees; LRT is the maximum Likelihood Ratio Test; the QTL effect is expressed in standard deviation units; the effect is given as Meishan - European alleles; +, *, ** are the 10%, 5% and 1% chromosome-wide significance levels respectively; the references of previously published data are given,/: when no previous result was available for this trait on the chromosome or when this QTL was not detected in the previous analysis.

### SSC2

Single-QTL detection analyses identified a chromosome-wide significant QTL affecting loin weight with the French design and a suggestive QTL affecting back fat thickness. These two QTL are located on the telomeric end of pig chromosome 2 (SCC2p) where the IGF2 gene is located and have already been described [6,8]. With the Dutch design, three chromosome-wide significant QTL affecting meat percentage, BFT and a* colour value were detected around position 25 cM, the latter two QTL already described in [11]. An additional suggestive QTL affecting the weight at weaning was also detected in the same region with the Dutch design. Combining the French and Dutch pedigrees, only two QTL reached the 5% chromosome-wide significance threshold: significant LRT values were obtained for a QTL influencing back fat thickness at position 1 cM and for a QTL affecting meat percentage at position 32 cM. Using multiple-QTL analyses no additional QTL was detected on SSC2. Additional analyses related to parent-of-origin effect were computed but are not reported in the present study.

### SSC4

**Figure 2:**
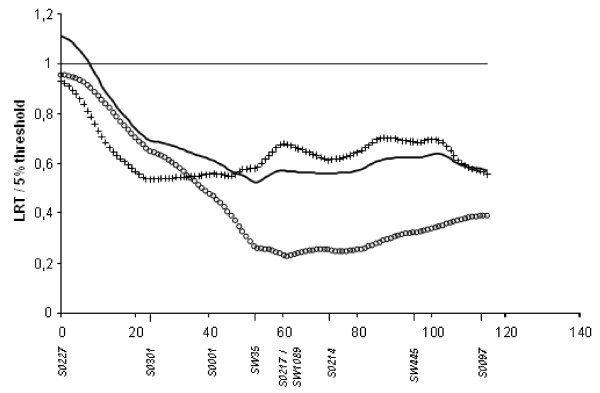
**QTL underlying Intra Muscular Fat content on SSC4 for the three studied pedigrees**. The solid line gives the result for the combined pedigree, the circled line for the French pedigree and the crossed line for the Dutch pedigree; for each analysis, the LRT is presented in proportion to the 5% threshold on the chromosome

**Figure 3:**
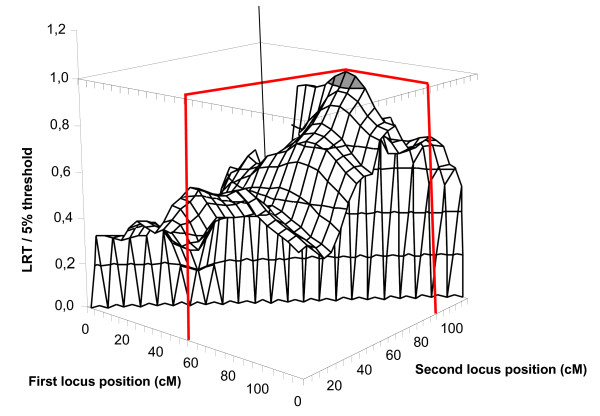
**Two-QTL analysis results for Life Weight Gain on SSC4 with the combined pedigree**. The z axis gives the value of the LRT divided by the 5% threshold at the chromosome-wide level, the surface shown in gray corresponding to a ratio higher than 1

With the French design on SSC4, two significant QTL affecting birth weight and belly weight around position 55 cM, and one QTL affecting life weight gain around position 66 cM were detected, all three QTL already described in [6,8]. Two additional suggestive QTL also previously described in [7] were identified affecting intra-muscular fat content at position 0 cM and back fat thickness at 61 cM [8]. Two new suggestive QTL were detected, affecting X2 at 14 cM, and head weight at 42 cM. Using multiple-QTL detection analyses, a pair of QTL localised at positions 30 cM and 74 cM was detected for teat number. With the Dutch design, only one QTL, previously described in [11] and affecting intra-muscular fat content at 0 cM reached the 10% chromosome-wide suggestive. When combining the French and Dutch pedigrees this QTL reached the 5% chromosome-wide significance threshold (Figure 2). A QTL affecting birth weight around position 55 cM, and a QTL affecting life weight gain at 83 cM, detected only with the French design, were also confirmed in the combined analysis. Using multiple-QTL tests, the hypothesis of two QTL affecting this trait was more likely than a single-QTL hypothesis: the test for loci at 59 and 90 cM reached the 5% chromosome-wide significance threshold (Figure 3). Additionally, a suggestive QTL influencing teat number was detected in the combined analysis at 46 cM using the single-QTL analysis. The two-QTL model retained in the analysis of the French pedigree for this trait was not significant with the combined analysis.

### SSC6

With the French design on SSC6, a significant QTL affecting midriff weight at 144 cM and two other suggestive QTL one influencing loin weight (99 cM) and one affecting teat number (110 cM) were detected. With the Dutch design, a significant QTL influencing L* parameter at 103 cM and two other suggestive QTL one affecting birth weight (136 cM) and one affecting teat number (154 cM) were identified. For this last trait, a two-QTL model was significant at the 5% chromosome-wide significance threshold for two loci localized at 50 and 155 cM. When combining the French and Dutch pedigrees only one significant QTL affecting teat number at 104 cM was detected at the 5% chromosome-wide threshold.

## Discussion

The aim of this study was to combine two F2 designs produced independently in France and in the Netherlands to detect QTL influencing economically important traits. These two designs were selected on the following criteria: (1) comparable founder breeds (Meishan and Large White and/or Landrace European breeds) and (2) the quasi-equal number of offspring produced in both protocols. Furthermore, although the European breeds were not identical, the Meishan sires used in the Dutch pedigree are related to the French Meishan dams. This supports the assumption that common Meishan QTL alleles segregated in both designs. European and Meishan breeds should have contrasted haplotypes (haplotypes being highly similar in both Meishan pedigrees) and QTL should segregate for the same loci. However, the two populations differ with respect to the number of families (six F1 sire families in the French design versus 39 F1 sires families in the Dutch design) and the reciprocal cross used to produce the F1 animals. To combine these designs the six French families and 24 of the 39 Dutch families, composed of more than 30 offspring, were retained. Our study focused on three pig chromosomes, SSC2, SSC4 and SSC6, for which QTL had already been detected. Despite the lack of overlap between some of the identified QTL from the pedigrees analysed separately, joint analyses of the combined pedigree should provide (1) greater significance of shared QTL, (2) exclusion of false suggestive QTL and (3) greater mapping precision for shared QTL. First, we investigated how the addition of new genotypes influenced the two designs. QTL detections were performed independently for each pedigree and for all the traits to be compared to the results previously published. We were then able to estimate the advantage between a combined analysis and independent analyses.

### QTL detected with the French design

In the analysis with the French design, the results were consistent with those previously reported although some differences were observed. In 2001, Bidanel et al. [6] have reported a highly significant QTL underlying BFT on SSC4 between two markers located at positions 43 and 83 cM. Addition of microsatellite SW35 at 52.7 cM in the present study resulted in a loss of significance for this trait (10% chromosome-wide threshold). On the opposite, we detected in the same region a suggestive QTL affecting head weight which had not been previously identified in this pedigree [8]. This shows that the addition of a single highly informative marker in a region with a low marker density can change results to a great extent. Cepica et al. [29] have reported a QTL affecting head weight in the same region on this chromosome. On SSC6, Milan et al. [8] have described a suggestive QTL for belly weight between positions 2 and 32 cM In our analysis, this QTL was neither significant, nor suggestive. In this case also, a microsatellite, SW2535, was also added above SW2406, which allowed a better coverage of the telomeric part of the chromosome. Due to the addition of this marker, we can conclude that the previously suggested QTL is probably a false-positive. Finally, with the French pedigree, three new QTL on SSC6: two QTL, one affecting loin weight (99 cM) and one affecting teat number (110 cM), and one significant QTL influencing midriff weight (144 cM). Previous studies had revealed QTL affecting loin weight on SSC6 at 83 cM in crosses involving Pietrain, Large-White and Leicoma animals [30], and between 122 and 149 cM with a Duroc x Pietrain design [31].

### QTL detected with the Dutch design

With the Dutch pedigree alone, it was possible that our results differed from those previously reported because of (1) the addition of new markers (as for the French pedigree), (2) the selection of 24 families among the 38 that were used by de Koning et al. [11] and (3) the model used (mixture of full and half-sib families vs. line cross model). Initially, de Koning et al. have detected many QTL regions using a line-cross model and our results are closer to those obtained using a half-sib model [10-12]. Among the previously QTL detected, we did not confirm two suggestive QTL influencing the b* colour value [10] and LWG on SSC6 [12]. These differences could be due to the addition of new markers showing that these QTL are false-positive ones. However, we cannot exclude that they were lost because of the reduction of the number of families. It is possible that these QTL were segregating in some of the 14 excluded families, which results in loss of power. However, with the addition of new markers in the Dutch pedigree, we detected a new QTL underlying the L* colour value on SSC6 at 103 cM. No QTL influencing this meat quality trait has been reported in this region. In a Landrace x Iberian design, Ovilo et al. [32] have described one QTL at 40 cM on SSC6 influencing meat lightness in interaction with another locus on SSC18. We also detected a QTL underlying birth weight at 136 cM while Yue et al. [33] had reported a QTL for the same trait in a cross between Meishan and Pietrain breeds at 98.2 cM.

Our results illustrate the influence of marker density to exclude false-positive QTL and to detect new ones. In 2008, Hu and Xu [34] have demonstrated using simulations that when the interval between two adjacent markers decreases, the power of QTL detection increases. Here we confirm this statement with real datasets.

### QTL detected combining the French and Dutch designs

**Figure 4:**
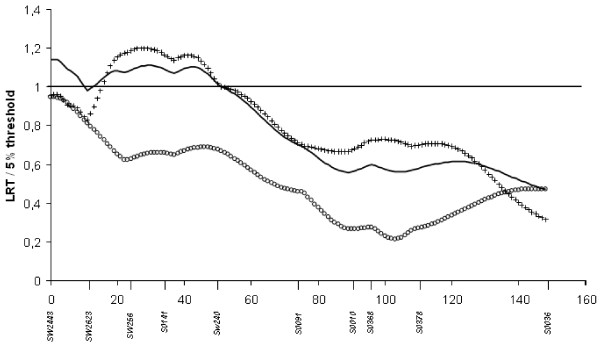
**QTL underlying BFT on SSC2 for the three studied pedigrees**. The solid line gives the result for the combined pedigree, the circled line for the French pedigree and the crossed line for the Dutch pedigree; for each analysis, the LRT is presented in proportion to the 5% threshold on the chromosome

The size of a QTL design has a major influence on the power of QTL detection. A QTL can be detected thanks to the recombination events occurring during the gamete production in F1 animals. In an F2 protocol, the number of recombination events is limited. Therefore, to confirm or refine the position of QTL initially detected with an F2 protocol, the number of crossing-over events cannot be increased without additional animals. Producing additional animals is expensive and time-consuming. Thus, a joint analysis of independent pedigrees is an easy alternative to enhance the number of F2 animals in the design. One suggestive QTL affecting intramuscular fat content on SSC4 had been detected in both the French and Dutch pedigrees independently. By combining the two designs, this QTL reached a 5% chromosome-wide significant threshold. As shown on Figure 2, the two pedigrees contributed equally to the LRT. The proportion of segregating fathers per pedigree and/or the QTL effect were probably too small to detect this QTL in each pedigree independently but the combined pedigree resulted in more power and the QTL reached the significance threshold. For SSC2, independent analyses of each pedigree indicated that a single QTL influencing BFT and meat percentage was segregating on the telomeric end of the short arm of this chromosome but not at the same position. With the French pedigree, LRT value was maximal in the IGF2 region (0 cM) whereas with the Dutch design it was around 28 cM. Analysis of the combined pedigree confirmed that in fact two linked loci are segregating in this region, i.e. the IGF2 gene and a locus located around 30 cM (Figure 4). QTL influencing meat percentage have been previously detected between 40 and 60 cM with crosses involving either Meishan, Pietrain and Wild Boar breeds [35], or Large White and Meishan animals [36].

Concerning the two-QTL detection analysis, our study provides evidence that combining two pedigrees and adding new markers increases the power of QTL detection. With a single-QTL analysis, we can conclude that there is a single QTL at 80 cM on SSC4 influencing life weight gain whereas in fact this maximum statistic is probably due to the combined actions of two QTL. The combination of two designs indicates that the presence of two different QTL located at 60 and 90 cM and common to both populations is more likely than the segregation of a unique QTL at 80 cM (Figure 3).

### Limits of the combined analysis

The benefit of combined analyses is essentially obtained when the QTL is segregating in both pedigrees. If QTL were segregating in only one of the two designs, detection power and estimated QTL effect could be reduced. We observed this situation for some QTL initially detected independently in one of both pedigrees. For example, a significant QTL affecting birth weight was detected only with the French design at 53 cM on SSC4 and was confirmed with the combined pedigree but it was not a major segregating QTL in the Dutch pedigree, the significance of this LRT was reduced in the combined analysis. Thus, this QTL is specific to the French pedigree. At the extreme, a QTL previously detected in one pedigree may be undetectable in the combined analysis. This situation was observed only for suggestive QTL such as that affecting birth weight on SSC6 detected in the Dutch pedigree only. Thus these QTL are also specific to the pedigree in which they were detected. The same situation has been reported by Walling et al. [4] who have shown that among seven different pedigrees a QTL affecting birth weight detected on SSC4 segregated in the French pedigree only but after adding the six other pedigrees, although the QTL was still detected, its significance was lower and its effect was divided by two.

### Influence of the breeds used

QTL segregating in several independent designs will be largely influenced by the breeds used. On the one hand, by combining two pedigrees (involving only three breeds), Kim et al. in 2005 [37] have detected 10 new QTL undetected in either of the two pedigrees. In this case, the combination of pedigrees increased the number of interesting regions. On the other hand, Pérez-Enciso et al. [5] in 2005 have demonstrated that by combining pedigrees, the possibility of detecting new QTL is sometimes reduced. This analysis was performed using five independent crosses involving six pig breeds (Iberian, Landrace, Large White, Meishan, Pietrain and Wild Boar). If a QTL allele is fixed in one breed involved in one cross only, the addition of other pedigrees that do not involve these breeds can reduce the effect of this QTL and therefore make it less detectable. This is also supported by the report of Guo et al. [38] in 2008 who have shown that if a QTL is population-dependant it is highly probable that combining pedigrees will provide no benefit. To avoid this drawback of a joint analysis, Guo et al. have combined two Meishan x Large White pedigrees with F0 animals coming from the same populations. Combining pedigrees seems more advantageous if pedigrees involve similar breeds like in our combined study. In our case, the breeds involved in both pedigrees were genetically very similar, which led to the detection of common QTL (BFT on SSC2 or IMF on SSC4). In some cases, the QTL detected with the combined pedigree had only been described in one of the two designs like birth weight on SSC4. This result can be due to either a population specific QTL, or to differences in the proportion of families contributing to the statistic signal.

### Reduction of QTL interval

An important advantage of a combined analysis is to estimate more precisely mapping intervals. This parameter may be largely influenced by the total number of markers used and the number of common markers analysed on the different designs. In our study, new data were generated by genotyping animals of both pedigrees with additional microsatellite markers to maximise the power and the precision. Previous studies that combined two or more pedigrees only analysed pre-existing datasets. The originality of our study is the large number of common markers, i.e. 29 common markers evenly spaced along the three chromosomes. In 2003, Bennewitz et al. [39] have combined two bovine granddaughter designs and surprisingly, obtained larger confidence intervals than with single designs. Previously, using simulations they had estimated that an increase of population size led to a reduction of confidence intervals [40,41]. Thus, they have proposed that the increase in confidence intervals is probably due to the fact that the families were genotyped with different sets of markers and only a few belonging to both sets. Using a dataset of highly common markers makes it possible to increase the confidence interval of a QTL. In our analysis, we avoided this drawback by analysing a unique set of microsatellite markers on both pedigrees and on the combined one. With a single set of markers, we obtained narrower confidence intervals than previously reported estimated intervals calculated with the same drop-off method [6,8]. In the present study, confidence intervals varied from 3 to 30 cM, with an average size of 15 cM. In the initial analyses, the confidence intervals spanned on average 40 cM. Unfortunately, since in the first analyses reported by de Koning et al. [11,12] no confidence intervals were calculated, we could not carry out a comparison.

## Conclusion

Combining two Meishan x European breeds F2 pedigrees improved the mapping of QTL compared to separate analyses of the pedigrees. We detected new QTL, confirmed some QTL which were previously described and excluded false positive QTL. Our work was facilitated by the access to raw phenotypic data and DNA of animals from both pedigrees. Analysis of a single set of markers proved more efficient to obtain smaller QTL intervals. The combination of two designs involving very similar breeds with the addition of new markers allowed us to fine-map QTL without phenotyping additional animals.

## Competing interests

The authors declare that they have no competing interests.

## Authors' contributions

FT carried out the genotyping and the QTL detection analyses. HG participated in the QTL detection analyses. HCMH provided the information and the phenotypic data sets of the Dutch pedigree. JPB provided the information and the phenotypic datasets of the French pedigree. MAMG and JR conceived of the study. JR participated in its design and coordination and helped to draft the manuscript. All authors contributed and approved the final manuscript.
